# A Potential Draft Sequence Analysis of Enterobacter asburiae Strain B6_18 of an Endophytic Bacterium

**DOI:** 10.1128/mra.01299-22

**Published:** 2023-04-05

**Authors:** Olubukola Oluranti Babalola, Victor Funso Agunbiade, Ayomide Emmanuel Fadiji

**Affiliations:** a Food Security and Safety Focus Area, Faculty of Natural and Agricultural Sciences, North-West University, Mmabatho, South Africa; University of Arizona

## Abstract

The work presented here describes the genomic analysis of the maize plant-isolated endophytic strain Enterobacter asburiae B6_18 from Northwest Province, South Africa, for potential maize plant growth-promoting traits.

## ANNOUNCEMENT

Plant endophytes are microorganisms that may colonize healthy plant tissues without doing any damage to the host plant and create a harmonious coexistence with the plant. They play a crucial role in the plant microecological system (1–3).

Root samples of maize (Zea mays L.) were collected from plantations at the teaching and research farm of the North-West University, South Africa (25° 47′ 25.24056″ S, 25° 37′ 8.17464″ E, 25° 47′ 30.14056″ S, 25° 37′ 9.27464″ E and 25.82080S: 025.61382E), cut into small pieces with a sterile scalpel, and washed in sterile distilled water. To ensure complete removal of the epiphytic bacteria, surface sterilization was achieved using the method adopted by Fadiji et al. (4). The plant material (1 g) was weighed, suspended in 1 M phosphate-buffered saline (PBS), and manually macerated in a mortar and pestle until a smooth suspension was obtained. Sample suspensions were serially diluted up to 10^−9^; and aliquots (0.1 mL) from dilutions 10^−5^ and 10^−6^ were pipetted into petri dishes and plated with sterilized LB agar. The inoculated petri dishes were incubated at 28°C for 24 h using the methods described by Babalola et al. (5). A pure endophytic bacterium culture of the bacterial isolate was obtained by repeated streaking onto sterile nutrient agar (NA) and incubation at 28°C for 24 h, as described by Ahmad et al. (6) and Majeed et al. (7). After subculturing, distinct bacterial colonies that had formed on the plates were counted and confirmed using morphological and biochemical tests. The isolate, Enterobacter asburiae strain B6_18, was observed to possess plant growth-promoting traits, such as phosphate solubilization (8) and the production of siderophore (9), indole-3-acetic acid, and ammonia (6). The discovery of these traits encouraged us to proceed with whole-genome sequencing of this strain.

Genomic DNA from the pure culture on solid agar was extracted using a Quick-DNA miniprep kit (Zymo Research, USA), following the manufacturer’s protocol for DNA extraction from fungi/bacteria. A sequencing library was prepared. Sequence reads were generated using the Illumina NextSeq 550 instrument (2 × 150 bp) at Inqaba Biotechnology Industries, South Africa, and the sequencing run yielded 6,938,274 bp of raw data. Using FastQC version 1.0.4 (10), the quality of the reads was examined. Trimmomatic version 1.2.14 was used to filter out the adapter regions and low-quality reads from the data (11). A genome assembly was created using SPAdes version 1.2.4 (12), yielding a total genome size of 4,532,593 bp with 27 contigs, 56.1% GC content, an *N*_50_ value of 449,685 bp, and an *L*_50_ value of 3. This sequence was then subjected to a BLAST search of GTDB-Tk version 1.7.0 (13). All software was run with default settings unless otherwise specified.

The genome assembly and annotation were performed using the KBase platform (14), and prediction of gene functions was carried out using the freely accessible NCBI Prokaryotic Genome Annotation Pipeline (PGAP) (15). The results revealed a total of 4,330 and 4,253 coding DNA sequences (CDSs), 31 pseudogenes, and 77 noncoding sequences (8 rRNAs, 64 tRNAs, and 5 noncoding RNAs [ncRNAs]).

### Data availability.

The whole-genome sequence of Enterobacter asburiae strain B6_18 has been deposited at DDBJ/ENA/GenBank under the accession number JAPFPX000000000. The version described in this paper is version JAPFPX010000000. The raw reads are available under the BioProject accession number PRJNA897876 and the BioSample accession number SAMN31582772; the sequence data obtained in this work have been deposited in the NCBI Sequence Read Archive under the accession number SRR22177482.
